# Conventional vs. diode laser stapedotomy: audiological outcomes and clinical safety

**DOI:** 10.1007/s00405-023-08429-4

**Published:** 2024-01-14

**Authors:** Leonardo Elías Ordóñez Ordóñez, Daniela Cerón Perdomo, Claudia Paola González Saboya, Felipe Osorio Mejía, Jorge Medina-Parra, Esther Sofía Angulo Martínez

**Affiliations:** 1 Department of Otolaryngology, Clínica Universitaria Colombia, Clínica Colsanitas SA, Keralty, Calle 23 #66-46, Bogotá, Colombia; 2 Department of Otolaryngology, Facultad de Medicina, Fundación Universitaria Sanitas (Unisanitas), Keralty, Bogotá, Colombia; 3 Hospital Militar Central, Universidad Militar Nueva Granada, Bogotá, Colombia; 4 Facultad de Medicina, Fundación Universitaria Sanitas (Unisanitas), Keralty, Bogotá, Colombia; 5 Anaboleas Research Team, Endorsed by Clinica Universitaria Colombia and Fundación Universitaria Sanitas (Unisanitas). Recognized by Colciencias (2021) Ministry of Science, Technology and Innovation of Colombia, Bogotá, Colombia

**Keywords:** Hearing loss, Otosclerosis, Surgery outcomes, Stapedotomy, Laser, Stapedectomy

## Abstract

**Purpose:**

To compare the hearing results and clinical safety of patients undergoing stapes surgery with conventional technique and diode laser.

**Methods:**

Retrospective observational study, which included patients treated with primary stapes surgery performed between January 2009 and January 2020. Three audiometric measurements (PTA, GAP and SDS) were evaluated as main results, evaluated by analysis of covariance (controlling the preoperative value). Intraoperative and postoperative complications were also analyzed. Outcomes were measured 6 months (± 1 month) after surgery.

**Results:**

153 cases were included, 97 operated with conventional technique and 56 with laser technique. Postoperative GAP ≤ 10 dB was obtained in 85.6% of the total sample, 82.5% in the conventional technique and 91.1% in the laser technique. Analysis of covariance showed no significant differences in the three surgery outcomes between the two groups (PTA, *p* = 0.277; GAP, *p* = 0.509 and SDS, *p* = 0.530). Regarding surgical complications, sensorineural damage was higher in the conventional technique group (*p* = 0.05). On the other hand, there were four cases of facial paresis, all in the laser group, three of them with the 980 nm laser.

**Conclusions:**

Stapedotomy offered a high percentage of hearing success in the two groups studied. There were no significant differences in audiometric result, but there was a differential presentation of complications, being more frequent sensorineural hearing loss in the conventional technique group and facial paresis in the laser group.

## Introduction

Otosclerosis is a disease of the bony labyrinth, due to a change in the composition and architecture of the otic capsule, which causes decreased mobility of the stapes footplate and progressive conductive hearing loss [1, 2]. Stapedotomy is the surgical technique of choice to treat conductive hearing loss caused by otosclerosis, its objective is to restore the movements of the ossicular chain with a prosthesis, after the removal of the stapes suprastructure and the opening of the perilymphatic space of the vestibule by perforating the stapes footplate [3].

Since the first description in 1876 by Johannes Kessel, who mistakenly considered that hearing loss was caused by increased fluid pressure in the inner ear [4], different techniques, steps and surgical instruments have been proposed to minimize the risk of injury to the inner ear: micro-instruments, micro drills and more recently lasers. These advances in technology to make the fenestra, cut of the stapes tendon and cut of the posterior crura seek to provide good hearing results while keeping the lowest possible percentage of complications [5].

A clinical audit study of results in Colombia showed that the hearing results of the conventional technique are comparable to those published internationally, but with a higher rate of complications [6]. Therefore, the authors suggested the introduction of techniques that reduce surgical trauma, such as the use of lasers [6]. Laser devices use ablation properties of a respective wavelength to generate defined cut lines and hemostasis [7]. They were introduced for the first time in otology by Sataloff, who used the laser on a scleral plaque in 1967, thus initiating the alternative of laser in stapedotomy [8].

Laser techniques have the advantage of decreasing bleeding, achieving greater accuracy, and may offer less damage to the inner ear and ossicular chain [9]. However, there is debate as to whether the laser technique offers better results than conventional cold methods. In a systematic review of the literature, it was found that there is no evidence of superior hearing results with the use of laser, compared to the conventional technique [9, 10]. However, there appears to be an increased risk of stapes footplate fracture and sensorineural hearing loss with conventional technique [9].

Studies have been published with different types of lasers, such as: argon, potassium-titanyl-phosphorous (KTP), CO_2_, and diode laser, without showing clear differences in the results between them [11–13]. Diode laser has the advantage of being contact/near-contact, with very good hemostatic effect and high precision for incisions [12, 13].

Although injury to the inner ear because of mechanical trauma may be less likely with the laser technique, other potentially deleterious effects such as thermal damage, heating of the perilymph and heating of neighboring structures such as the facial nerve should not be neglected [9]. To compare the hearing and clinical safety outcomes of patients undergoing stapes surgery with conventional and laser techniques, the following study was conducted.

## Materials and methods

This is a retrospective observational study. The stapes surgeries were performed by the same surgeon in the otorhinolaryngology service of a third-level referral center in Bogotá, Colombia. Patients who underwent surgery from January 2009 to January 2020 were included.

### Patients

All patients who underwent primary stapes surgery for otosclerosis or congenital stapes fixation over 18 years of age were included. Patients in whom stapes footplate fixation was not confirmed during the surgical procedure, those with tympanosclerosis, who required ossicular chain reconstruction, and those with incomplete information were excluded.

Eligible patients were identified through the surgical scheduling files of the study institution. Information was obtained retrospectively from the electronic medical record. For data collection, a digital instrument was created to record demographic and clinical information, relevant data from the surgical description, intraoperative complications, postoperative complications and audiological tests performed before and after surgery. The patients included in the study are all the patients who underwent stapes surgery during the selected time interval.

### Evaluation of the auditory result

The auditory outcomes were evaluated through the pure tone average (PTA) of 4 frequencies (0.5–3 kHz), the difference of audiometric thresholds (GAP = PTA air conduction–PTA bone conduction) and the percentage of speech discrimination score (SDS). For each of the three results, preoperative results were compared with postoperative results taken 6 months (± 1 month) after surgery.

In addition, surgical success was categorized following the guidelines for reporting results in stapes surgery of the American Academy of Otolaryngology–Head and Neck Surgery [14]. An excellent result was defined when the postoperative GAP was ≤ 10 dB, a good result when the postoperative GAP was ≤ 20 dB, and a poor result when the postoperative GAP was > 20 dB.

### Clinical safety

The main clinical safety outcome was established as the assessment of sensorineural damage. This was defined as a postoperative drop in four-frequency bone conduction PTA ≥ 15 dB and/or drop in SDS ≥ 15%.

In addition, complications presented intraoperatively and postoperatively are described, along with relevant findings that could have been related to the event during the surgical procedure.

### Technical characteristics of the laser

In the surgical procedures in which laser was used, two types of equipment (different wavelengths) were used sequentially, according to the technological availability of the supplier. Initially, the 980 nm diode laser was available (from April 2016 until September 2018, called "red" laser) and subsequently the 445 nm diode laser (October 2018 until the end of the study, called "blue" laser) (A.R.C. Laser GmbH, Nϋrnberg, Germany). Table 1 shows the parameters of use of the equipment.

**Table 1:** Parameters of use of the 980 nm and 445 nm diode lasers

Laser type/Anatomical site of use	Laser parameters		
	Power (W)	Pulse duration (ms)	Relaxation time (ms)
980 nm Laser			
Cutting the tendon of the stapes muscle	2.0	100	700
Vaporization of the posterior crura	3.5	100	700
Fenestration of the stapes footplate	3.0	100	700
445 nm Laser			
Cutting the tendon of the stapes muscle	4.0	100	500
Vaporization of the posterior crura	4.0	100	500
Fenestration of the stapes footplate	2.0	100	400

*nm* nanometers, *W* Watts, *ms* miliseconds

### Surgical technique

**Fig. 1:**
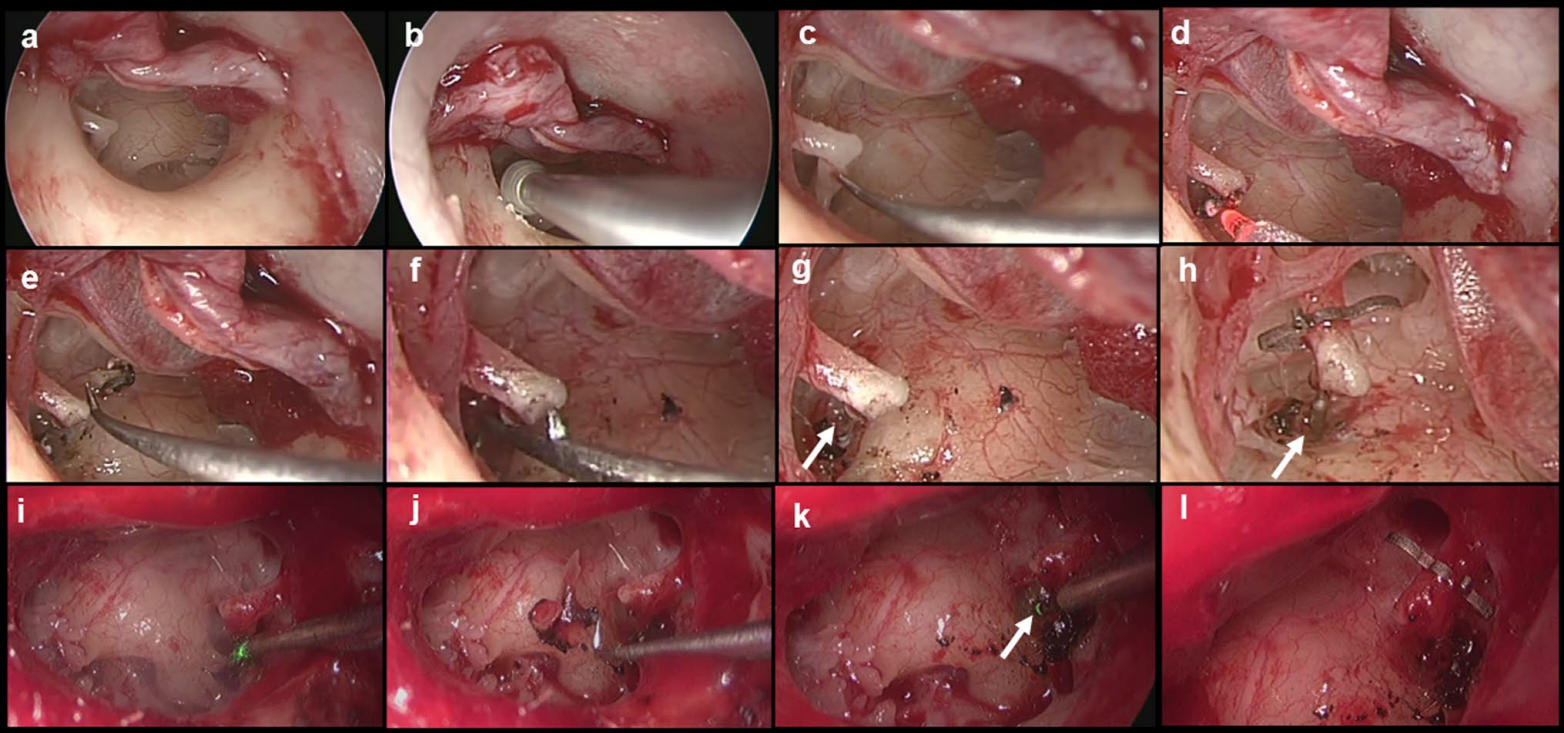
Surgical technique of laser stapedotomy. Stapedotomy with laser technique, right ear intervened with 980 nm laser (**a**–**h**) and left ear with 445 nm laser (**i**–**l**). Transcanally, the tympanomeatal flap is elevated to expose the middle ear (**a**) and the scutum is reamed with a diamond bur (**b**). This step can be done alternatively with a bone curette. Then, intraoperative confirmation of otosclerosis is made: mobile hammer and incus with stapes fixed by sclerotic plates on the  footplate (**c**). The tendon of the stapes muscle is cut and the posterior crura is vaporized with the diode laser fiber (**d**). The incudostapedial joint is then disarticulated and the anterior crura is fractured inferiorly with a fine 45° or 90° hook (**e**). The distance between the footplate of the stapes and the lateral face of the incus (**f**) is measured; 250 µm is added to this distance to select the length of the prosthesis. A 500 µm window is made with the laser in the middle third of the footplate (white arrow), to use a 400 µm piston (**g**). Then, the prosthesis is placed, in this case àWengen titanium piston-clip (**h**), verifying that the piston is embedded in the fenestra (white arrow). Note how in the laser technique the surgeon first cuts the tendon of the stapes muscle and vaporizes the posterior crura (**i**), before disarticulating the incudostapedial joint; modification with respect to the initial technique described by Shelton, then proceeding to fracture the anterior crura (**j**). Since the diode laser has a malleable handpiece and works by contact, it allows the surgeon great flexibility in its handling, such as when making the fenestra (white arrow) on the platinum (**k**). The àWengen titanium clip-piston prosthesis is manually anchored, automatically closed and allows 180° of the circumference of the incus to be preserved without pressure on the vascular supply (**l**)

All patients in this study were operated by the same surgeon, using uniform surgical techniques; this reduces the variability resulting from different surgeons/surgical techniques and learning curve, helping to control biases due to these confounding factors. The treating surgeon prefers to perform stapedotomy whenever possible and only performs stapedectomy when stapes footplate fracture is present intraoperatively, Fig. 1**.**

The techniques used are a modification of the stapedotomy described by Shelton [15]. Briefly, under general anesthesia and by transcanal approach, the skin of the external auditory canal (EAC) is incised in its bony portion and the tympanomeatal flap is elevated until reaching the tympanic annulus. The scutum is drilled and/or curetted and the motility of the three ossicles of the ossicular chain is evaluated. When otosclerosis is confirmed (fixation of the stapes by sclerotic plaques at the level of the footplate, with mobile malleus and incus), the tendon of the stapes muscle and the posterior crura are cut/vaporized, the incudo-stapedial joint is disarticulated and then the supra-structure of the stapes is fractured inferiorly, exposing the entire footplate. The distance between the footplate and the lateral face of the incus lenticular is measured and the length and type of prosthesis is defined. Subsequently, the fenestra is made on the stapes footplate, in the conventional technique it is done with a manual perforator or micro drill (Osseostap. Bien-Air Surgery SA, Swiss), while in the laser technique, it is vaporized in the center of the footplate, trying to make (in both cases) a fenestra of ± 500 µm. The prosthesis is then placed, anchored to the incus and the fenestra is closed with a hematic patch. The prosthesis of choice (surgeon's preference), due to its versatility for placement, automatic closure and 180° anchorage, which leaves another 180° without compromising the vascular supply to the lenticular process of the incus, was the àWengen titanium clip-piston (Heinz Kurz Medizintechnik GmbH, Germany). In cases where the diameter of the long process of the incus was seen to be larger than average (> 1 mm), a titanium K-piston prosthesis (Heinz Kurz Medizintechnik GmbH, Germany) was selected, which unlike the clip-piston requires manual closure of the loop over the long process of the incus [6]. As of April 2017, when there was technological availability of prostheses made of nitinol (nickel–titanium alloy, heat-activated shape memory metal), this prosthesis (Nitibond; Heinz Kurz Medizintechnik GmbH, Germany) was used as an option to the titanium clip-piston.

When fracture of the stapes footplate causing a floating segment of the same occurred, the surgery was transformed to a stapedectomy: the footplate remnants were removed with a straight hook, a temporal fascia graft was taken to close the oval window, and the prosthesis was placed and the procedure continued as described above.

### Statistical analysis

Quantitative variables were described by measures of central tendency and dispersion, while categorical variables were presented with frequencies and percentages. For proportional/interval variables, the normality of their distribution was tested using the Kolmogorov–Smirnov test; those with normal distribution were analyzed using parametric tests.

Analysis of covariance (ANCOVA) was performed to compare the main outcomes between the results of the two groups (conventional technique vs. laser technique) and repeated measures *t* tests for comparison of measurements before and after surgery in each group. For categorical variables, Chi-square test and Fisher's exact test were used, according to the expected count in the contingency tables. Statistical significance was defined as *p* < 0.05 (two-tailed test) and the analyses were performed in SPSS software (v.11.5; SPSS, Inc., Chicago, IL, USA).

This study meets the ethical standards established by the Helsinki declaration [16], the Colombian resolution 8430 of 1993 for studies in human beings [17] and has the approval of the Institutional Ethics and Research Committee (CEIFUS 687–20 act No. 019-20). For the reporting of this study, we followed the standards for reporting observational studies: STROBE ("The Strengthening the Reporting of Observational Studies in Epidemiology") [18]. No data that could identify an individual patient were recorded, thus guaranteeing the confidentiality and privacy of the study subjects, in accordance with national regulations.

## Results

**Fig. 2:**
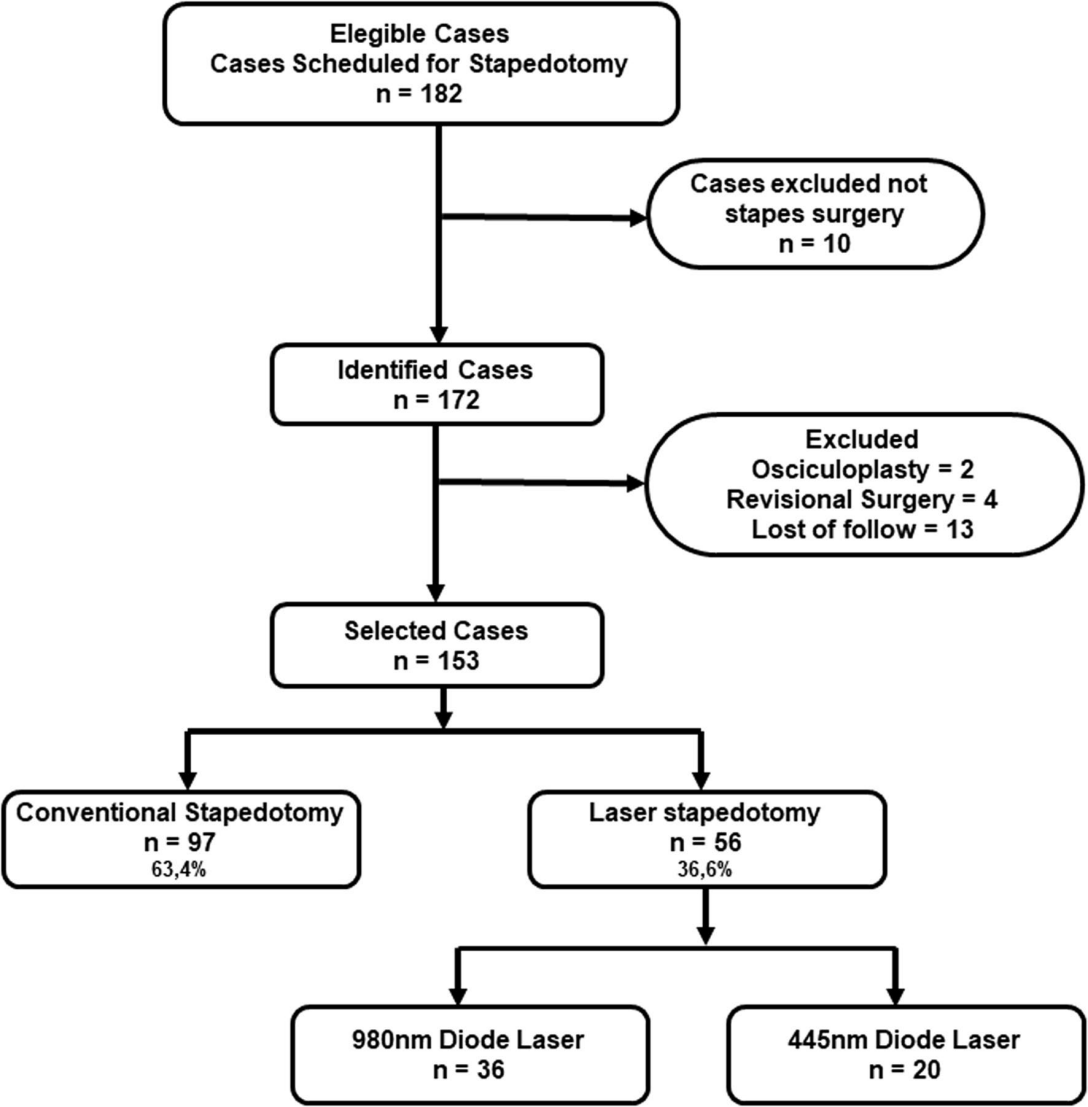
Flow chart of the study patients

A total of 182 eligible cases were found, of which 172 met the inclusion criteria and 153 were selected for the final analysis, Fig. 2**.** Out of the 19 cases of losses, 13 cases were excluded due to incomplete follow-up, four because they were revisional surgeries and two because stapes surgery was not performed but osciculoplasty was performed due to intraoperative findings.

Table 2 shows the demographic and clinical characteristics of the patients in the study. The mean age at the time of surgery was 44.1 ± 11.17 years, most patients were women 92 (60.1%) and in all cases the surgical indication was the diagnosis of otosclerosis (there were no cases of congenital stapes fixation). In 106 cases (69.3%), bilateral disease was present and in 79 cases (52%) surgery was performed on the left side. In the initial phase of the study period, when laser was not available, 97 surgeries (63.4%) were performed, with conventional cold technique (until March 2016). Later with the availability of the 980 nm diode laser, 36 surgeries were performed (23.5%) and then with the availability of the 445 nm diode laser, 20 cases (13.1%) were operated; for a total of 56 cases (36.6%) operated with laser. The most used prosthesis was the titanium clip piston in 118 cases (77.1%), followed by the nitinol prosthesis in 22 cases (14.4%) and the titanium K-piston in 12 cases (7.8%).

**Table 2:** Clinical and demographic characteristics of the patients

Variables	Type of surgery		Total (*n* = 153, %)
	Conventional stapedotomy (*n* = 97)	Laser stapedotomy (*n* = 56)	
Gender			
Female	63	29	92 (60%)
Male	34	27	61 (40%)
Age (years)			
*x* ± ST	43.92 ± 11.37	44.32 ± 10.93	44.1 ± 11.17
Affected side			
Right	17	6	23 (15%)
Left	8	16	24 (16%)
Bilateral	72	34	106 (69%)
Surgery side			
Right	52	22	74 (48%)
Left	45	34	79 (52%)
Type of prosthesis			
Titanium Clip piston	86	32	118 (77%)
Titanium K-piston	10	2	12 (8%)
Nitinol prosthesis	0	22	22 (14.4%)
Other	1	0	1 (0.6%)
Prosthesis length (mm)			
4.0	60	15	75 (49%)
5.0	25	31	56 (36.6%)
4.5	10	9	19 (12.4%)
3.5	1	0	1 (0.7%)
4.25	1	0	1 (0.7%)
5.5	0	1	1 (0.7%)

*ST* standard deviation

The audiological results are shown in Table 3**,** where the preoperative, postoperative values and differences are presented for the conventional technique and laser technique groups. Since the three selected postoperative outcome variables (PTA, GAP and SDS) have respective values in the preoperative period, an analysis of covariance (ANCOVA) was performed to make comparisons. To use the preoperative values as covariable, a linear regression analysis was first performed between the preoperative (covariable) and postoperative (dependent variable) values for the three outcomes: PTA, GAP and SDS. The values were confirmed to have a linear relationship and were statistically significant: PTA (*r* = 0.587, *p* < 0.001), GAP (*r* = 0.249, *p* = 0.002) and SDS (*r* = 0.451, *p* < 0.001). Problems of collinearity were also ruled out, because no Pearson correlation coefficient was > 0.7. It was found that there were no statistically significant differences between the conventional technique and the laser technique for any of the three outcome variables: PTA (*p* = 0.277), GAP (*p* = 0.509) and SDS (*p* = 0.530); ANCOVA. There were also no differences in preoperative baseline between the two surgical technique groups.

**Table 3:** Audiological outcomes in postoperative and preoperative groups

Variables	Type of surgery		Difference between groups *p*
	Conventional stapedotomy (*n* = 97)	Laser stapedotomy (*n* = 56)	
PTA (dB), Mean ± ST			
Preoperative	53.9 ± 11.4	54.3 ± 9.9	– 0.4 (IC95% = – 3.9 to 3.2) 0.842*
Postoperative	28.0 ± 11.4	29.8 ± 9.2	– 1.8 (IC95% = – 5.3 to 1.7) 0.277**
GAP (dB), Mean ± ST			
Preoperative	30.3 ± 7.8	31.9 ± 8.9	– 1.7 (IC95% = – 4.4 to 1.1) 0.227*
Postoperative	7.15 ± 6.99	6.79 ± 5.51	0.37 (IC95% = – 1.8 to 2.5) 0.509**
SDS (%), Media ± ST			
Preoperative	98.8 ± 5.1	100 ± 0	– 1.2 (IC95% = – 2.6 to 0.14) 0.081*
Postoperative	99.20 ± 4.5	100 ± 0	– 0.80 (IC95% = – 1.9 to 0.4) 0.530**

*PTA*: pure tone average of four frequencies (0.5–3 kHz), *dB* decibels, *ST* standard deviation, *GAP* = PTA air conduction–PTA bone conduction, *SDS* Speech discrimination score

^*^Student´s *t* test. It was used to rule out differences in the preoperative baseline in the three outcomes

^**^Analysis of Covariance (ANCOVA): was used to compare differences in postoperative outcomes between the two surgical techniques. The dependent variable was used in the model: postoperative outcome; independent variable-fixed factor: type of surgery; covariate: preoperative outcome

Regarding the auditory outcomes within each group (differences in), it was found that there was a statistically significant difference between postoperative and preoperative in both the conventional surgery group and the laser surgery group for PTA and GAP.﻿ For the conventional technique, PTA (*p* < 0.001), GAP (*p* < 0.001) and SDS (*p* = 0.438) were found; while in the laser technique group PTA (*p* < 0.001) and GAP (*p* < 0.001); repeated measures *t* tests. No differences could be calculated in the laser technique group for SDS between postoperative and preoperative, as the values were constant. Table 3 shows the mean and standard deviation for each measurement within the group, it is observed that in the preoperative baseline there were no statistically significant differences between the two groups (*p* = 0.842, *p* = 0.227 and *p* = 0.081; respectively). In the main outcomes, there were no statistically significant differences between the two groups for any of the three audiological measurements in the postoperative period (*p* > 0.05).

Table 4 categorizes the postoperative results according to the recommendations of the American Academy of Otolaryngology and Surgery of Head and Neck Surgery [14]. The surgical outcome was excellent in 131 patients (85.6%), of which 80 (82.5% = 80/97) were performed by conventional technique and 51 (91.1% = 51/56) with laser technique. Regarding the type of laser used, the result was excellent in 32 (88.9% = 32/36) cases operated with 980 nm laser and in 19 (95% = 19/20) cases with 445 nm laser. In accordance with the ANCOVA results, there was no statistically significant difference in the results between the conventional technique vs. laser technique (*p* = 0.361).

**Table 4:** Hearing outcomes according to the surgical technique

Hearing outcome^a^	Tipo de cirugía		*p* value
	Conventional stapedotom y (*n* = 97)	Laser stapedotomy (*n* = 56)	
Excellent			
*n* (%)	80 (82.47%)	51 (91.07%)	0.361**
Good			
*n* (%)	95 (97.94%)	56 (100%)	
Bad			
*n* (%)	2 (2.06%)	0 (0%)	

^**^Fisher`s exact test

^a^Results according to the classification suggested by the American Academy of Otolaryngology and Head and Neck Surgery

In relation to the clinical safety of surgery, there were five (3.3% = 5/153) patients with intraoperative complications. One case (0.65%) had gusher (cerebrospinal fluid fistula through the inner ear), two patients (1.31%) had floating footplate and two patients (1.31%) had laceration of the tympanic membrane. The gusher case was controlled with prosthesis positioning and the use of temporal fascia grafting around the fenestra. In the cases of floating footplate, the fragments of the footplate were removed, temporal fascia graft was placed over the oval window (the surgery was transformed into stapedectomy) and the prosthesis was coupled. In one of the cases of tympanic membrane laceration, temporal fascia graft was used and in the other one, closure by second intention was left, there was no postoperative tympanic perforation in either of the two cases. Regarding postoperative complications, they are presented in Table 5**,** showing that there were no complications in 92.2% (141/153) of the cases. The most frequent complication was vertigo, in 4.6% (7/153); followed by facial paresis in 2.6% (4/153) of the cases. The cases of vertigo received medical management (dimenhydrinate in the acute phase plus vestibular therapy), with symptomatic improvement. The four cases of facial paresis had partial compromise of nerve function (GII to GV, House–Brackmann scale) and was presented between the fifth and 12th postoperative day (late paresis). All four patients received a treatment of corticosteroid (prednisolone 1 mg/Kg/d) for 7 days and physical therapy; with complete recovery of their facial motility (House–Brackmann I) within 4 weeks postoperatively.

**Table 5:** Postoperative complications

Type of complication	Conventional stapedotomy (*n* = 97)	Laser stapedotomy (*n* = 56)
Vertigo		
*n* (%)	6 (6.2%)	1 (1.8%)
Surgical wound infection		
*n* (%)	1 (1%)	0
Facial nerve paresis		
*n* (%)	0	4 (7.1%)
Sensorineural hearing loss		
*n* (%)	16 (16.5%)	2 (3.9%)
None		
*n* (%)	90 (92.8%)	51 (91.1%)

Since some intraoperative and postoperative complications are clearly not associated with the type of technique (since clinically they are associated with the identical steps of the two techniques), a consolidated analysis was made of those that may occur as a result of the technique used; to improve the discrimination of the analysis. The case of "gusher" was excluded, because it is related to an anatomical defect that causes a greater communication between the internal auditory canal (IAC) and/or cochlear aqueduct and the perilymphatic space. The two cases of tympanic membrane laceration were excluded, because this occurs during tympanomeatal flap elevation, a step that is identical in the two techniques. The case of surgical wound infection was excluded, because it is not expected to be related to the use or non-use of the laser, which is used in the intermediate of the surgery, temporarily distant from the approach and closure of the incision. Thus, there were a total of 12 cases with intraoperative/postoperative complications (7.8% = 12/153); due to the fact that one case with floating footplate presented postoperative vertigo at the same time. Although there were no statistically significant differences between the surgical techniques used with respect to clustered complications, *p* = *0.358*, Fisher's exact test; the four cases of facial paresis occurred with the laser technique (three with 980 nm laser and one with 445 nm laser), the two cases of floating footplate were with 980 nm diode laser and six cases of vertigo occurred with the conventional technique (the other case was with 980 nm laser). There was only one complication with the use of the 445 nm laser in the clustered analysis of complications (1/20 = 5%).

Regarding sensorineural hearing loss, it occurred in 18 cases (11.8% = 18/153), 16 cases with the conventional technique and 2 cases with laser; *p* = 0.05, Fisher's exact test. The two cases of sensorineural damage in the laser technique occurred with the 980 nm laser.

## Discussion

The study shows that the results achieved in terms of hearing success are similar between the conventional technique and the laser technique, controlling for presurgical values. These results are congruent with those reported in a clinical trial and a systematic review on this specific aspect of surgical outcome [9, 12]. Stapedotomy and stapedectomy are surgical procedures with a high success rate: > 80% for an excellent outcome [6, 9, 12] and are considered the treatment of choice in patients with otosclerosis or congenital stapes fixation [3, 4]. Although some surgeons prefer stapedectomy (complete resection of the stapes footplate with complete exposure of the oval window), over the years stapedotomy (small fenestra) has gained acceptance and is now considered the technique of choice for exposing the inner ear in stapes surgery [5, 6].

Laser stapedotomy is a technique that has been proposed for several decades [8], although it has not shown a clear advantage in success rate over the conventional technique [9–12, 19–21], has been associated with a lower frequency of complications. A systematic review on the use of laser reports a higher success rate with this technology [5]. However, this is a systematic review that used descriptive studies as input, which, therefore, cannot be considered a study with a high level of evidence on the subject. On the other hand, in a clinical trial (Pradipta et al. 2016), they report a postoperative GAP of 10.86 ± 5.4 dB for 30 patients operated with conventional technique and 11.05 ± 5.3 dB for a group of 30 patients operated with diode laser; difference that was not statistically significant (*p* > 0.05) [12]. In our study, GAP closure < 10 dB is reported in 91.07% of laser-operated patients vs 82.47% of patients operated with conventional technique, a non-statistically significant difference (*p* = 0.361). A previous clinical audit study of results, in the same institution, where this study was carried out and with conventional technique, had shown similar results in terms of surgical success with respect to international reports [6].

The diode laser has been used in stapes surgery and some of its advantages are easy manipulation and adaptation to the narrow spaces of the middle ear [7, 12]. At the study institution, 980 nm and 445 nm diode lasers were available sequentially, which allowed us to review some outcomes separately for each type of technology. The hearing outcomes were similar in the three groups (no laser/980 nm laser/445 nm laser) and comparable to those described in the literature [5, 12, 18], with a differential trend among the three groups with respect to intraoperative and postoperative complications. In the laser group there was lower frequency of vertigo and sensorineural hearing loss, reaching the limit of statistical significance for sensorineural damage (*p* = 0.05). This may be related to the lower mobilization of the stapes footplate, which decreases the risk of inner ear lesions, as suggested by Motta et al. [20]. In that study they point out that conventional techniques presented greater difficulties in adjusting the diameter of the fenestra to the caliber of the prosthesis. Since the use of laser offers better hemostasis, it may improve the visibility of the surgical field [5, 7, 9, 11, 13, 19] and thus decrease the number of complications. Our study, however, also showed a higher frequency of late facial paresis in the group that underwent laser surgery: all four patients who presented it were in this group. Although all four cases improved with medical management, facial paresis is a complication that generates a high degree of anxiety and concern among patients and surgeons. The risks of thermal damage using laser, which does not occur with the conventional technique, have been warned and is an aspect that surgeons should consider if they use this technology [11]. Late facial paresis, that one which does not occur in the immediate postoperative period and, therefore, is not related to direct intraoperative trauma to the facial nerve, has been described as an infrequent event following stapes surgery and with a favorable prognosis [22]. Ng and Maceri reported two cases of facial paresis in laser stapedotomy, suggesting that when this technique is used, it may increase the incidence of facial paralysis due to the heating of the tympanic cord that could cause retrograde edema of the main trunk of the facial nerve [23]. Although the cause of neural dysfunction has not been clearly established in these cases, different mechanisms such as surgical stress, intraoperative trauma, dehiscent fallopian canal, and reactivation of latent viruses (varicella zoster, herpes simplex virus types 1 or 2; or Epstein Barr virus) have been proposed in relation to the thermal effect of the laser [24]. The four cases of facial paresis presented in this study were late (5–12 days postoperatively) and with complete recovery in the first month after the onset of the condition, a clinical course similar to Bell's palsy. Of the four cases, three were operated with 980 nm laser, which has a higher degree of tissue penetration (and, therefore, energy dispersion) than 445 nm laser, it may have a greater potential for thermal trauma to the facial nerve [7, 12, 13]. The use of 445 nm diode laser, due to its lower tissue penetration [13], seems to decrease these risks according to the data of this study, although due to the small number of patients with complications, this cannot be definitively established. Therefore, to reduce the risk of thermal trauma to the tissues, we recommend using the laser in stapes surgery with the lowest power necessary for the specific surgical step and favoring the intraoperative cooling of the middle ear: increasing the ratio time (period between laser shots) and decreasing the duration of its pulse (do not use continuous mode) (see Table 1).

Among the limitations of the study, there were no randomized groups that would allow for analyses with greater statistical power. Neither was it possible to evaluate other variables that may affect the surgical outcomes, such as the type of prosthesis and the type of laser technology (due to time availability). In addition, although the evaluation of outcomes at 6 months postoperatively is an adequate period for the analysis of sensorineural hearing loss damage and other complications of surgery, it should be considered an intermediate time for the evaluation of surgical success. Among the strengths of the study, we sought to control for confounding bias by including stapes surgeries performed by a single surgeon with uniform techniques. In addition, the use of preoperative audiometric values as a covariable in assessing postoperative hearing outcome increases statistical power and reduces biases due to variability in the audiometric baseline. The results of this study can guide the decision making of surgeons when they are going to perform stapedotomies and have the option of using the diode laser.

In conclusion, the results found show that stapedotomy is a procedure with a very good success rate both with conventional and laser techniques. Although there were no differences in the success rate, the laser technique tended to show a lower frequency of complications, such as sensorineural damage and postoperative vertigo. However, caution should be exercised when using laser in stapes surgery, since complications related to thermal damage to tissues can occur, such as facial nerve paresis, which was more frequent. The use of laser sources with lower energy dispersion, such as the 445 nm diode laser, could increase safety with the use of this technology.

## Data Availability

The authors stated that all information provided in this article could be shared and the raw data can be requested by email to the corresponding author.
